# Low‐Temperature Charge/Discharge of Rechargeable Battery Realized by Intercalation Pseudocapacitive Behavior

**DOI:** 10.1002/advs.202000196

**Published:** 2020-06-10

**Authors:** Xiaoli Dong, Yang Yang, Bingliang Wang, Yongjie Cao, Nan Wang, Panlong Li, Yonggang Wang, Yongyao Xia

**Affiliations:** ^1^ Department of Chemistry and Shanghai Key Laboratory of Molecular Catalysis and Innovative Materials Institute of New Energy iChEM (Collaborative Innovation Center of Chemistry for Energy Materials) Fudan University Shanghai 200433 P. R. China

**Keywords:** high diffusion coefficient, intercalation pseudocapacitance, rechargeable batteries, synergistic effects, ultralow temperature

## Abstract

Conventional intercalation compounds for lithium‐ion batteries (LIBs) suffer from rapid capacity fading and are even unable to charge–discharge with temperature decline, owing to the sluggish kinetics and solvation/desolvation process. In this work, a high‐performance rechargeable battery at ultralow temperature is developed by employing a nanosized Ni‐based Prussian blue (NiHCF) cathode. The battery delivers a high capacity retention of 89% (low temperature of −50 °C) and 82% (ultralow temperature of −70 °C) compared with that at +25 °C. Various characterizations and electrochemical investigations, including operando Fourier transform infrared spectra, in situ X‐ray diffraction, cyclic voltammetry response, and galvanostatic intermittent titration technique are carried out to detect the structural stability and electrochemical behavior at different temperatures. It turns out that the pseudocapacitive behavior drives the desolvation process at the interface, while fast diffusion in the bulk electrode accelerates the movement of Li^+^ from the interface to the bulk materials. The unique synergistic features of intercalation pseudocapacitance at the electrolyte/electrode interface and high diffusion coefficient in the bulk electrode enables the NiHCF cathode with excellent low temperature performance. These findings offer a new direction for the design of LIBs operated at low temperature.

## Graphical abstract

Benefitting from the synergistic effect of pseudocapacitive behavior at the interface and high diffusion coefficient in the bulk electrode, a rechargeable battery with excellent low temperature performance is realized with nanosized Ni‐based Prussian blue as cathode, exhibiting a high capacity retention of 89% at −50 °C and 82% at ultralow temperature of −70 °C compared with that at +25 °C.

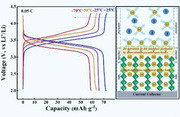

Commercialized lithium‐ion batteries (LIBs) have occupied widespread energy storage market, but still encountered the poor performance at low temperature,^[^ ^1^, ^2^, ^3^, ^4^, ^5^ ^]^ which greatly limits the practical applications under extreme conditions such as high‐altitude areas and aerospace explorations. This can mainly be attributed to three factors: the increased viscosity and reduced ionic conductivity of electrolyte; the sluggish desolvation and the enlarged resistance of the electrode/electrolyte interface; and slow solid‐state diffusion process of Li‐ions into the bulk electrodes.^[^ ^6^, ^7^, ^8^ ^]^ Generally, the intercalation of Li^+^ experienced the following stages: First, solvated Li‐ions migrate from liquid electrolyte to the electrode surface, which corresponds to the ionic conductivity of the electrolyte. Second, solvated Li‐ions occur desolvation and across the electrode/electrolyte interface, relating with the desolvation process. Third, desolvated Li‐ions migrate through the solid electrolyte interphase layer and then into the electrode, in concert with solid electrolyte interphase (SEI) resistance and solid‐state diffusion process in the bulk material. All these processes would be impacted by the temperature, especially when the temperature declines to subzero. To date, certain achievements have been accomplished through developing electrolytes with enhanced ionic conductivity,^[^ ^2^, ^3^, ^9^, ^10^, ^11^, ^12^ ^]^ modifying the SEI for promoted Li^+^ mobility,^[^ ^8^, ^12^, ^13^ ^]^ and so on. In spite of the improved low temperature discharge behavior, corresponding recharge procedures at low temperature are still very difficult,^[^ ^2^, ^3^, ^8^, ^9^, ^10^, ^11^, ^12^, ^13^, ^14^, ^15^ ^]^ Organic polymers showed well charge and discharge performance at ultralow temperature of −70 °C,^[^ ^4^, ^6^ ^]^ but still encountered low energy density owing to their inherent low potential and tapping density. Commonly used intercalation compounds electrodes, such as olivine‐type LiFePO_4_, layered oxides LiCoO_2_, LiNi_1−_ *_x_*_−_ *_y_*Co*_x_*Mn*_y_*O_2_ (NCM), LiNi_1−_ *_x_*_−_ *_y_*Co*_x_*Al*_y_*O_2_ (NCA), and spinel‐type LiMn_2_O_4_, LiNi_0.5_Mn_1.5_O_4_, etc., generally exhibit high potential and high specific energy. Nevertheless, the sluggish desolvation process at the interface and slow diffusion in the bulk electrodes lead to the failure of LIBs at low temperature.^[^ ^16^, ^17^, ^18^, ^19^ ^]^ Therefore, it is urgently desired to develop electrode materials that could charge at low temperature. Prussian blue and its analogues (PBAs, A*_x_*M[Fe(CN)_6_]*_y_*·□_1−_ *_y_*·*z*H_2_O, A represents mobile cations and M represents transition metal cations) have been well known as a large family of transition‐metal hexacyanoferrates with open framework structure.^[^ ^20^, ^21^, ^22^, ^23^, ^24^, ^25^ ^]^ The distinctive feature of PBAs structure enables generous spacing of 3D diffusion channels, attributed to weak interaction with moving ions that facilitate its inward and outward transport. Benefitting from the large interstitial A sites and roomy channels of PBAs lattice, the reversible insertion of cations is available from three dimensions, thus exhibits a higher ionic conduction than that of conventional intercalation compounds.^[^ ^21^ ^]^ The unique characteristics of such kind of intercalation materials are likely to promote LIBs operated at low temperature, which has not been explored until now.

Herein, we demonstrated a rechargeable lithium battery based on nanosized NiFe‐PBA [NiHCF for short, HCF: hexacyanoferrate, Fe(CN)_6_] as cathode and metallic lithium anode, which exhibited excellent charge/discharge performance at low temperature. The open ionic channels and broad interstitial spaces in the 3D framework were demonstrated via XRD investigation. Operando Fourier transform infrared (FTIR) spectra clearly detected the reversible changes in the cyanide stretching during the oxidation and reduction process. Galvanostatic intermittent titration technique (GITT) was carried out to determine the diffusion coefficient, which turns out to be 10^−9^ cm^2^ s^−1^ at +25 °C and achieves as high as 10^−10^ cm^2^ s^−1^ at −70 °C. Benefitting from the open framework structure and nanosized morphology, the surface‐controlled intercalation pseudocapacitive behavior was ascertained with cyclic voltammetry (CV) investigations, which promoted the desolvation process at the interface layer. Enabled by these unique characteristics, NiHCF‐based battery exhibited a high capacity retention of 89% at low temperature of −50 °C, and still as high as 82% at an even lower temperature of −70 °C, demonstrating excellent low temperature performance.

NiHCF was synthesized through a facile wet‐chemical synthetic method.^[^ ^26^ ^]^ As revealed with inductively coupled plasma atomic emission spectroscopy (ICP‐AES) and elemental analysis, the ratio of Na, Ni, Fe, C, N can be given as 1.57:1:0.86:5.16:5.16. The crystal structure was identified with XRD (**Figure** **1**a), from which the as‐prepared NiHCF powder exhibits a monoclinic phase, similar to the reported monoclinic Na_2_Mn[Fe(CN)_6_]·1.87H_2_O and Na_2_Mn_2_(CN)_6_·2H_2_O with a space group of *P2_1_/n*.^[^ ^27^, ^28^ ^]^ Rietveld refinement of the powder XRD pattern indicated that the proposed monoclinic structure fits well for the NiHCF material, with favorably low refinement reliability factors (*R*_wp_ = 7.81%, *R*_p_ = 4.43%, *χ* ^2^ = 1.768%). The lattice parameters can be obtained as *a* = 10.241(4) Å, *b* = 7.369(2) Å, *c* = 7.122(3) Å, and *β* = 91.18(3)° (Table S1, Supporting Information, for detailed atomic parameters). Accordingly, the local structure was schematically illustrated in Figure 1b. The cell volume was 537.35(34) Å^3^, with the C—N—Ni angles of 148.37° and 170.18°, different from a typical 180° in cubic phase.^[25]^ The corresponding distances of Ni⋅⋅⋅Fe are 5.1241 and 5.1205 Å. Each unit cell consists of eight subunit cells, thus contains eight large interstitial sites (4.6 Å), that can host mobile ions, such as Li^+^, Na^+^, and zeolitic water. As elucidated by field emission scanning electron microscopy (FESEM) image in Figure 1c, NiHCF exhibited a granular morphology with an average size of about 20–30 nm, in well correspondence with transmission electron microscopy (TEM) image (Figure S1, Supporting Information). The nanosize ensured the effective electrolyte infiltration and shorten the transport distance for ions, which can compensate the reduced diffusion coefficient of the electrode material at low temperature. The Brunauer–Emmett–Teller (BET) results further displayed a distinct pore size distribution centered at about 6 nm with a surface area of 57.6 m^2^ g^−1^ (Figure S2, Supporting Information), which indicated the formation of internal open channels. Additionally, open crystal structure is conducive to the migration of mobile ions and could effectively accommodate the lattice volume variation. FTIR spectrum showed a strong absorption peak at 2080 cm^−1^ (Figure 1d), which is attributable to stretching vibration of the bridging —C≡N— ligands coordinated to Fe^II^. The weak band at 3360 and 1606 cm^−1^ corresponded to the bending of H—O—H and stretching of O—H from interstitial water, respectively. Besides, the characteristic mode at 608 cm^−1^ was associated with —Fe—CN— in‐plane deformation. Thermogravimetric analysis (TG) and differential scanning calorimetry (DSC) experiment were introduced to determine the water content in the terminal formula, as shown in Figure 1e. The entire weight loss is 13% with temperature increasing to 250 °C, which attributed to be the deprivation of water from PB structure. The water loss comes from two parts, one is the adsorbed water that weakly bonded on the surface or stored in interstitial sites (4.5 wt%, below 150 °C), the other is the coordinated water (8.5 wt%, 150–240 °C). When the temperature was raised to around 300 °C, NiHCF framework would start to decompose and liberate the (C≡N)_2_ units from the skeleton. In light of above comprehensive analysis, the chemical formula for prepared NiHCF can thus be determined as Na_1.57_Ni[Fe(CN)_6_]_0.86_·□_0.14_·1.5H_2_O, indicating a low content of Fe(CN)_6_ vacancies and coordinated H_2_O in the framework.

**Figure 1:**
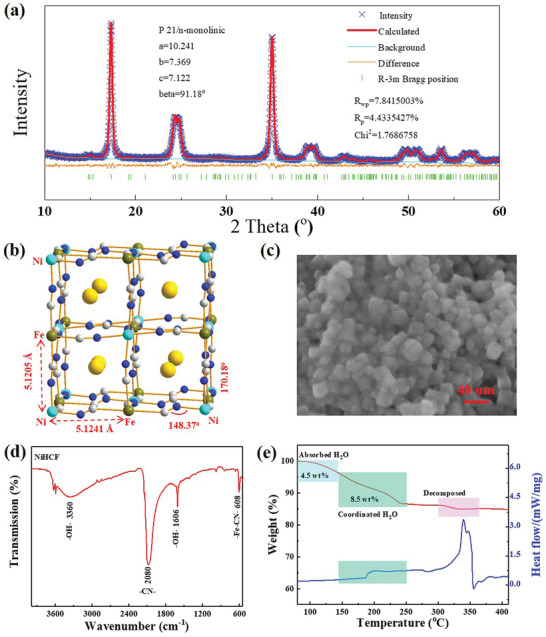
Materials characterizations. a) Rietveld refinement of the powder XRD pattern; b) illustration of cell structure and lattice parameter from the refinement; c) FESEM image; d) FTIR spectra with KBr pellet; and e) TG and DSC analysis under N_2_ atmosphere.

In order to probe the behavior of NiHCF with the insertion/extraction of Li^+^, in situ characterizations coupled with electrochemical investigations were carried out. As shown in **Figure** **2**a, a sandwich two‐electrode configuration spectra‐electrochemical cell (Figure S3, Supporting Information) was introduced to record the CV response for in situ Fourier transform infrared attenuated total reflection (FTIR/ATR) spectroscopy. Its validity was further evaluated through the comparison with coin cell at same scan rate of 0.2 mV s^−1^ (Figure S4, Supporting Information). The well‐consistent single pair of redox peaks (3.43 V vs Li^+^/Li) indicated the redox‐active site of C‐coordinated Fe^II^, which is close to the equilibrium voltage of the [Fe^II^(CN)_6_]^4−^/ [Fe^III^(CN)_6_]^3−^ redox couple (3.39 V vs Li^+^/Li). To ensure the high sensitivity, difference spectra were collected from FTIR spectroscopy. The frequency of the cyanide vibration stretching mode (2080 cm^−1^) is sensitive to the oxidation state of iron, which were illustrated as the contour map in Figure 2b and the difference spectra in Figure S5 (Supporting Information). The highly reversible changes of −CN− stretching mode upon oxidation and reduction can be clearly detected. The −CN− band became weakening and slightly shift to high wavenumber, along with the Fe^II^(CN)_6_ being oxidized to Fe^III^(CN)_6_. Similar trend can also be detected from the varieties of C═O bond in the EA solvent molecule (Figure S6, Supporting Information), indicating the corresponded reversible solvation/desolvation processes. In situ XRD was applied to detect the structural change of NiHCF upon varying Li^+^ content during charge/discharge conducted by controlling the capacity with a constant current of 0.1C. As shown in Figure 2c, the crystal framework was well remained without any structural phase transition upon Li^+^ insertion/extraction. This can be attributed to the electrochemical inertness of Ni^2+^ in the skeleton and corresponding zero‐strain insertion characteristics. What's more, the shift of XRD peaks is almost negligible at various charge/discharge states, which well indicated the superior structural stability of the rigid open framework skeleton.^[^ ^29^ ^]^ It has also been reported that nanostructuring materials can suppress crystallographic phase transitions by decreasing intercalation stress. Besides, the unchanged lattice structure also implied that the redox reactions are likely confined to the surface of the material,^[^ ^30^ ^]^ which would be further proved with the latter kinetics investigation of electrode reactions.

**Figure 2:**
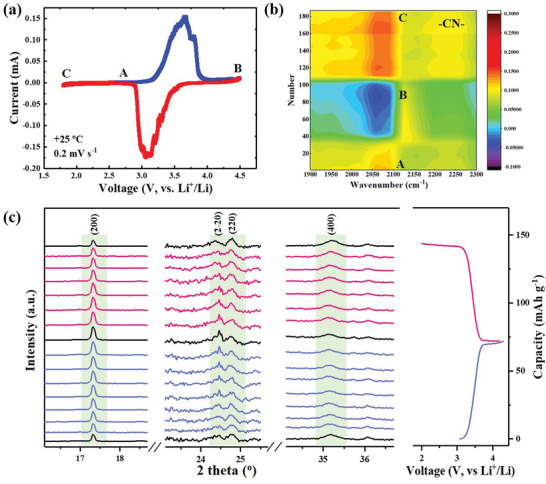
In situ analysis of mechanism. a) CV curve obtained with the spectra‐electrochemical cell and b) corresponding contour map of the reversible cyanide vibration stretching during the charge/discharge through operando FTIR analysis; c) in situ XRD patterns of the NiHCF cathode at different states during first cycle.

The good reversibility and structural stability offer advantages for NiHCF as cathode material. Considering its 3D open framework with large interstitial channels and nanoparticulate morphology, NiHCF should allow rapid Li^+^ diffusion in the electrode material. In order to reveal the feasibility of NiHCF as a candidate for low‐temperature LIB, the solid‐state diffusion of Li^+^ was investigated through GITT. As presented in **Figure** **3**a, GITT was carried out at a pulse of 0.1C rate within the potential from 2.0 to 4.3 V versus Li^+^/Li. The corresponding apparent diffusion coefficient of Li^+^ (*D*_Li_) upon the insertion/extraction in the host framework can be effectively determined based on the Fick's second law of diffusion (Figure 3b, refer to Figure S7, Supporting Information, for details). At room temperature, the *D*_Li_ ranged from 10^−9^ to 10^−8^ cm^2^ s^−1^ during the lithiation and delithiation processes, which corresponds well with the reported NiHCF.^[^ ^31^, ^32^ ^]^ It should be noted that the solid‐state diffusion of Li^+^ acts as a bottleneck for insertion‐type materials during the charge storage process, especially at low temperature. The high *D*_Li_ enables the fast solid‐state diffusion of Li^+^ in bulk NiHCF, which circumvents the limiting rate of commonly used intercalation compounds (LMO, LFP, etc.).^[^ ^16^, ^17^, ^18^, ^19^ ^]^ To dig out the ion migration kinetics, a CV experiment was carried out within a variation of sweep. A thin‐film electrode was used for 0.05–2 mV s^−1^, and a cavity microelectrode was used for 5–2000 mV s^−1^ so to alleviate the loss of ohmic polarization (Figure S8, Supporting Information). In theory, the voltammetric response obeys a power‐law relationship of measured current (*I*, mA) with the sweep rate (*v*, mV s^−1^)

(1) $$\begin{array}{c} i=av^{b} \end{array}$$

For a typical intercalation process limited by semi‐infinite linear diffusion, the peak current *i* varies with *v* ^1/2^ (i.e., *b* = 0.5); for a surface‐controlled capacitive process, it varies with *v* (i.e., *b* = 1). Accordingly, the *b*‐value of NiHCF in the range of 0.05–2 mV s^−1^ can be determined as 0.81 for cathodic and 0.80 for anodic peaks (Figure 3c,d), respectively, indicating that the kinetics are primarily surface‐controlled intercalation pseudocapacitive behavior, and thus fast.^[^ ^33^ ^]^ A change in the slope occurs at the scan rates higher than 5 mV s^−1^, corresponding to a decrease in *b*‐value to 0.57 for cathodic and 0.58 for anodic currents (Figure S9, Supporting Information). The limitation at higher scan rates can be attributed to kinds of factors including diffusion constraints and increased ohmic resistance from both active material and solid‐electrolyte interphase, similar to the reported intercalation electrodes T‐Nb_2_O_5_ and TiO_2_.^[^ ^30^ ^]^ The quantitative capacitive contribution can be determined from the total current response (detailed calculation can be referred to the Supporting Information). Figure 3e shows that proportions of the capacitive‐controlled capacity at the scan rate of 2 mV s^−1^, in which the contribution percentage was up to 93.2%. The proportion of the surface‐capacitance‐controlled contribution in total was increased from 66.0% (0.05 mV s^−1^) to 93.2% (2 mV s^−1^) (Figure 3f), implying that the Li^+^ storage kinetics is not controlled by the semi‐infinite diffusion process. Another representative characteristic is that the peak shift is smaller than 0.25 V at sweep rates below 20 mV s^−1^ (Figure S10, Supporting Information), which also identifies the intercalation pseudocapacitive behavior of the monoclinic NiHCF. Such feature can help overcome the sluggish solid‐diffusion process in the electrolyte/electrode interface, which is often considered as one of the impeding factors for the operation of LIBs at low temperature.

**Figure 3:**
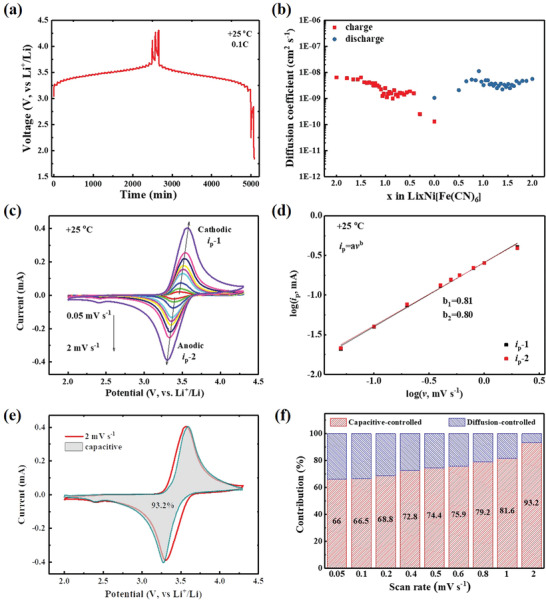
Kinetics analysis of lithium storage for the NiHCF electrode. a) GITT curves at 0.1C rate and b) the calculated apparent diffusion coefficient of Li^+^ upon lithiation/delithiation. c) CV responses with various scan rate from 0.05 to 2 mV s^−1^ and d) the determination of the *b*‐value according to log(*v*)–log(*i*_p_) relationship. e) Contribution of the capacitive and diffusion process at a scan rate of 2 mV s^−1^. f) Contribution ratios of the capacitive‐controlled process at different scan rates.

Benefitting from the unique properties of high diffusion coefficient and intercalation pseudocapacitive behavior, NiHCF might display good charge–discharge performance at low temperature. To verify this point, the electrochemical performance and structural changes of the NiHCF cathode at different temperatures were investigated in details, presented as **Figure** **4**a. A discharge capacity of 72 mAh g^−1^ was obtained with the 0.05C rate at room temperature (+25 °C), nearly reached the theoretical capacity of monoactive reaction. When the temperature is reduced to subzero, the battery can deliver a capacity of 66 mAh g^−1^ at −25 °C, corresponding to a high capacity retention of 92% (66 mAh g^−1^/72 mAh g^−1^ = 92%). With the temperature further decreasing to −50 °C, a reversible capacity of 64 mAh g^−1^ can still be obtained with 89% of that at +25 °C. At an even lower temperature of −70 °C, the capacity can still be kept at 59 mAh g^−1^, exhibiting an incredibly high capacity retention of 82%. Electrochemical impedance spectroscopy (EIS) investigation indicated the small increase of resistance, which resulted in the little polarization at low temperature (Figure S11, Supporting Information). Additionally, capacity retention with various C‐rates at different temperatures was also compared in Figure 4b and Figure S12 (Supporting Information), from which fast charge–discharge ability at low temperature can be detected. In order to give a better insight, the comparison with recent reports about low temperature performance was listed as Table S2 (Supporting Information), where present work shows superiority in the capacity retention over the reports. The fast kinetics can also be demonstrated with the CV analysis at −70 °C (Figure S13, Supporting Information). This significantly outperformed that of conventional intercalation compounds, which cannot work well at such low temperature (Figure S14, Supporting Information). Ex situ XRD investigations revealed almost identical patterns without any change (Figure S15, Supporting Information), both at room temperature (+25 °C) and at low temperature (−70 °C). This well indicated the superior stability of the open‐framework structure, conforming to the in situ XRD characterization in Figure 2c. The extremely small structural change upon lithiation/delithiation together with the open transport pathways of the crystalline framework is key for the operation at low temperature. Meanwhile, the excellent performance at low temperature can be partially attributed to the high diffusion coefficient of NiHCF cathode. Even at −70 °C, the apparent *D*_Li_ still ranges from 10^−11^ to 10^−9^ cm^2^ s^−1^ (Figures S16 and S17, Supporting Information). At the mid‐voltage of 3.43 V, the *D*_Li_ was about 10^−10^ cm^2^ s^−1^, which is an order of magnitude lower than +25 °C (Figure 4c). According to the proportional relationship of component diffusion coefficient (*D*_KLi_) with temperature (Please refer to the calculations in the Supporting Information for details), the reduced *D*_Li_ can be attributed to the increased resistance of electrode/electrolyte layer at low temperature.^[^ ^8^, ^13^ ^]^ The lithiation process of NiHCF cathode was illustrated as Figure 4d. The pseudocapacitive feature promoted the desolvation process of Li^+^ from electrolyte/electrode interface and the fast ions diffusion accelerated the migration of Li ions in the bulk electrode. The synergistic effect of high diffusion coefficient and surface‐controlled intercalation pseudocapacitive behavior turn out to be a perfect combination, which greatly facilitates NiHCF with the excellent performance at low temperature.

**Figure 4:**
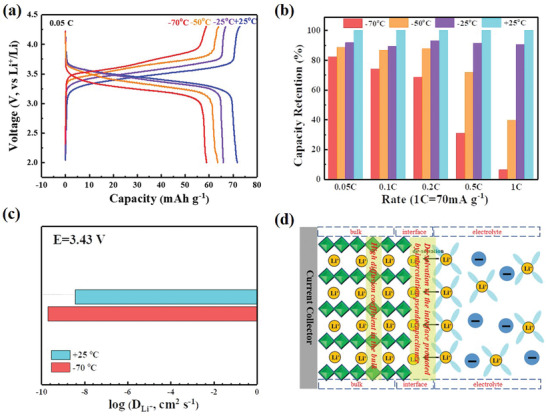
Electrochemical performance of NiHCF‐based LIB at different temperatures. a) Charge/discharge profiles with the rate of 0.05C from +25 to −70 °C. b) Comparison of rate performance. c) The variety of *D*_Li_^+^ between room temperature and ultralow temperature of −70 °C. d) Schematic illustration of lithiation process with the synergistic effect of surface‐controlled intercalation pseudocapacitive behavior at the interface and high diffusion coefficient in the bulk electrode.

In conclusion, nanosized NiHCF with open framework structure was prepared to demonstrate a high‐performance LIB at ultralow temperature of −70 °C. Benefitting from its open ionic channels and large interstitial spaces, NiHCF exhibited a high diffusion coefficient (≈10^−9^ cm^2^ s^−1^ at +25 °C and ≈10^−10^ cm^2^ s^−1^ at −70 °C) and intercalation pseudocapacitive behavior, which facilitates the operation of LIBs at low temperature. Operando FTIR spectra revealed the high reversibility of Fe−C−N−Ni, and in situ XRD proved the retention of the 3D rigid framework upon charge/discharge. Owing to its unique features, NiHCF based LIB exhibited an incredibly high capacity retention of 89% at −50 °C and 82% at −70 °C compared with that at +25 °C. The results provide a guidance for more discovery of electrode candidates to develop LIBs at low temperature.

## Conflict of Interest

The authors declare no conflict of interest.

## Supporting information

Supporting Information
