# Supplementary material for: Low‐Temperature Charge/Discharge of Rechargeable Battery Realized by Intercalation Pseudocapacitive Behavior
Source: Adv Sci (Weinh). 2020 Jun 10;7(14):2000196. doi: 10.1002/advs.202000196 (PMC7375234; doi:10.1002/advs.202000196)
Supplement: Supplementary file 1 — Supporting Information [file ADVS-7-2000196-s001.pdf]

Copyright WILEY-VCH Verlag GmbH & Co. KGaA, 69469 Weinheim, Germany, 2018.

Supporting Information

Low-temperature Charge/discharge of Rechargeable Battery Realized by Intercalation Pseudocapacitive Behavior

Xiaoli Dong, Yang Yang, Bingliang Wang, Yongjie Cao, Nan Wang, Panlong Li, Yonggang Wang, Yongyao Xia**

Dr. X. L. Dong, Y. Y. Yang, B. L. Wang, Y. J. Cao, N. Wang, P. L. Li, Prof. Y. G. Wang, Prof. Y. Y. Xia
Department of Chemistry and Shanghai Key Laboratory of Molecular Catalysis and Innovative Materials, Institute of New Energy, iChEM (Collaborative Innovation Center of Chemistry for Energy Materials), Fudan University, Shanghai 200433, P. R. China

E-mail: xldong@fudan.edu.cn; yyxia@fudan.edu.cn

Experimental Methods

Preparation of material

Prussian blue NiHCF powder was prepared via a facile citrate-assisted controlled crystallization method according to previous report. [26] In the procedure, 1 mmol $NiCl_2$ and 1 mmol trisodium citrate were dissolved into 20 mL deionized H_2O to form solution A. Solution B was obtained with 1 mmol $Na_4Fe(CN)_6$ in 20 mL deionized H_2O. Then, solution A and solution B were simultaneously added drop by drop into 100 mL deionized H_2O. The coprecipitation reaction went on under magnetic stirring for 12 hours after complete addition. The solution became cloudy gradually. The precipitate was centrifuged and washed with H_2O for three times, then dried at 100 ^oC overnight in a vacuum oven.

Characterizations of material

The crystal structure of as-prepared NiHCF was characterized with XRD on a powder diffractometer (Bruker D8 Advance, Germany) equipped with a Cu-Ka radiation (λ=0.15406 nm). The obtained data were analyzed using the Rietveld method of refinement of GSAS software. The compositions of the obtained NiHCF powder were determined through

inductively coupled plasma emission spectroscopy (ICP-AES, PE-8000) for Na, Ni and Fe elements. The morphology and particle size were recorded by field emission scanning electron microscope (FE-SEM s-4800) and transmission electron microscopy (JEOL 2100F). EDS analysis attached to SEM was applied to further confirm the elements composition and distribution in the NiHCF material. Thermogravimetric measurement was processed to detect water contents in the NiHCF material with a heating rate of 10 ^{o}C/min under N_2 flow (TGA, 209F1D-0053-L). The Brunauer-Emmett-Teller (BET) method was applied to investigate the specific surface area and pore volume. The quenched solid density functional theory (QSDFT) method was utilized to acquired pore size distribution.

Electrochemical investigations

The electrode was prepared by mixing active materials, carbon black and polyvinyl difluorides with the weight ratio of 7:2:1. The mixed slurry was then cast onto an aluminum foil and dried under vacuum at 80°C for 24 hours. The mass loading of active materials is about 1~2 mg cm^{-2}. The electrochemical data were collected by electrochemical workstation and charge/discharge instruments. The applied electrolyte was 5 mol kg^{-1} Lithium bis(trifluorosulfonyl)imide (LiTFSI) in ethyl aceatate (EA) diluted with dichloromethane (DCM) (1:4 by volume), named as 5m-1-4. The compatibility of the 5m-1-4 electrolyte with Li-metal anode was demonstrated in our previous work. [6] In brief, the electrolyte displayed a stable electrochemical potential window of 0~4.85 V (vs. Li^{+}/Li), low freezing point of -104.4^{o}C, high ionic conductivity of 1 mS cm^{-1} at -40°C and 0.6 mS cm^{-1} at -70°C, ensuring its feasibility at low temperature. Electrochemical tests including charge/discharge performance and GITT were performed on a Landt instrument (Wuhan, China) controlled by a computer. A high-low temperature test chamber (**Figure S17**), whose operating temperature range is -75°C – 100 ^{o}C, was used to provide a constant temperature environment for the investigations at various low temperatures. All the batteries were kept at a specific temperature for at least 2 hours to reach equilibrium of the battery temperature and the chamber pre-set temperature.

CV investigations were carried out with a three-electrode configuration cell and cavity micro-electrode (**Figure S8**).

In-situ FT-IR analysis

FT-IR measurements were carried out with the NICOLET 6700 FT-IR Spectrometer with a thermally stable DTGS detector cooled with liquid nitrogen. The signal was obtained by averaging 264 scans at resolution of 4 cm^{-1}. The applied range was from 4000 to 650 cm^{-1}. To ensure the sensitivity of the in-situ FT-IR spectra, difference spectra were recorded after oxidation or reduction process by subtracting the spectrum from the initial one (background) at open circuit voltage. Electrochemical experiments were carried out in a sandwich configuration cell depicted as **Figure S3**. HZ-7000 was utilized as the electrochemical workstation for cyclic voltammetric (CV) investigation. Scan rate was set as 0.4 mV s^{-1} between the voltage window of 2.0~4.3 V (vs. Li^+/Li).

The calculation of apparent diffusion coefficient D_{Li}

GITT was carried out at a pulse of 0.1C-rate within the potential from 2.0 to 4.3 V vs Li^+/Li. Schematics of a single step of the GITT was illustrated in **Figure S7**, during which a constant current charge/discharge for 20 min (t) was followed with an interruption of 60 min between each pulse to allow full relaxation at the open circuit potential (E_0) and reach a stabilized potential value (E_s). ΔE_t shows the potential change during the constant current pulse and ΔE_s represents the steady-state potential change between each interruption step. According to the Fick's second law of diffusion:

$$D_{Li} = \frac{4}{\pi}\left(\frac{m_B V_m}{M_B S}\right)^2 \left(\frac{\Delta E_s}{t(\frac{dE_t}{d\sqrt{t}})}\right)^2 \quad \left(t \ll \frac{L^2}{D_{Li}}\right) \qquad \text{[Equation 1]}$$

In which m_B, L, S are the mass, thickness and surface area of electrode, respectively. M_B is the molecular weight and V_m is molar volume.

If E vs. $\sqrt{t}$ is linear over the entire titration period t, the equation can be simplified when

$$D_{Li} = \frac{4}{\pi t}\left(\frac{m_B V_m}{M_B S}\right)^2 \left(\frac{\Delta E_s}{\Delta E_t}\right)^2 \quad \left(t \ll \frac{L^2}{D_{Li}}\right)$$ [Equation 2]

The component diffusion coefficient can be expressed as following equation:

$$D_{KLi} = -\frac{4kT m_B V_m I_o}{\pi c_A q^2 z_A^2 M_B S^2 t}\left(\frac{\Delta E_s}{t\left(\frac{dE_t}{d\sqrt{t}}\right)}\right)^2 \quad \left(t \ll \frac{L^2}{D_{Li}}\right)$$ [Equation 3]

Similarly, if E vs. $\sqrt{t}$ is linear over the entire titration period t, the component diffusion coefficient may also be determined from:

$$D_{KLi} = -\frac{4kT m_B V_m I_o}{\pi c_A q^2 z_A^2 M_B S^2}\left(\frac{\Delta E_s}{\Delta E_t}\right)^2 \quad \left(t \ll \frac{L^2}{D_{Li}}\right)$$ [Equation 4]

This means that the component diffusion coefficient D_{KLi} is in direct proportion to the temperature T. Therefore, the value of D_{KLi} is 1.47 times higher at room temperature (+25°C) than that at low temperature (-70°C).

The determination of capacitive contribution

In theory, the voltammetric response obeys a power-law relationship of measured current (*I*, mA) with the sweep rate (v, mV s^{-1}):

$$i = av^b$$ [Equation 5]

$$\log i = \log a + b \log v$$ [Equation 6]

in which the b value is the parameter to evaluate the electrode process. For a typical intercalation process limited by semi-infinite linear diffusion, the peak current i varies with $v^{1/2}$ (i.e. b=0.5); for a surface-controlled capacitive process, it varies with v (i.e. b=1).

The capacitive contribution can be quantified by separating the current response i at a fixed potential V into capacitive reactions ($k_1 v$) and diffusion-controlled process ($k_2 v^{1/2}$) with following equation:

$$i(V) = k_1 v + k_2 v^{1/2}$$ [Equation 7]

$$i(V)\, v^{-1/2} = k_1 v^{1/2} + k_2$$ [Equation 8]

where k_1 and k_2 are adjustable parameters. Accordingly, the values can be obtained from the line fitting of $i(V)\ v^{-1/2}$ versus $v^{1/2}$, and the capacitive contribution could be calculated out at each specific potential, which was shown in **Figure 3f**.

Table S1. Atomic parameters from Rietveld XRD

Atom	Wyck.	S.O.F.	x/a	y/b	z/c	U [Å^2]
Ni1	2a	1	0.5	0.5	0.5	0.0097(1)
Fe1	2d	1	0.5	0	0	0.0037(1)
C1	4e	1	0.4832(5)	0.1659(5)	0.7986(7)	0.0095(5)
C2	4e	1	0.1986(2)	0.4908(8)	0.5211(7)	0.0094(7)
C3	4e	1	0.5156(3)	0.2400(9)	0.2291(3)	0.0095(6)
N1	4e	1	0.4822(4)	0.3088(7)	0.7202(9)	0.0096(6)
N2	4e	1	0.3075(3)	0.4775(1)	0.5126(8)	0.0266(5)
N3	4e	1	0.4801(6)	0.3286(1)	0.2991(2)	0.0094(8)
O1	4e	1	0.2491(2)	0.1642(4)	0.2839(6)	0.2114(4)
Na	4e	1	0.2652(8)	0.5129(5)	0.9740(4)	0.0050(9)

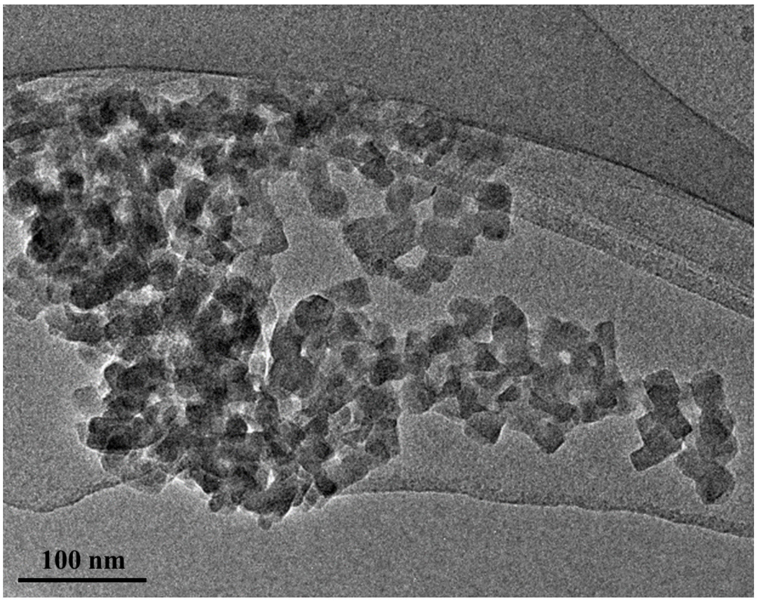

Figure S1. TEM of the as-prepared NiHCF powder.

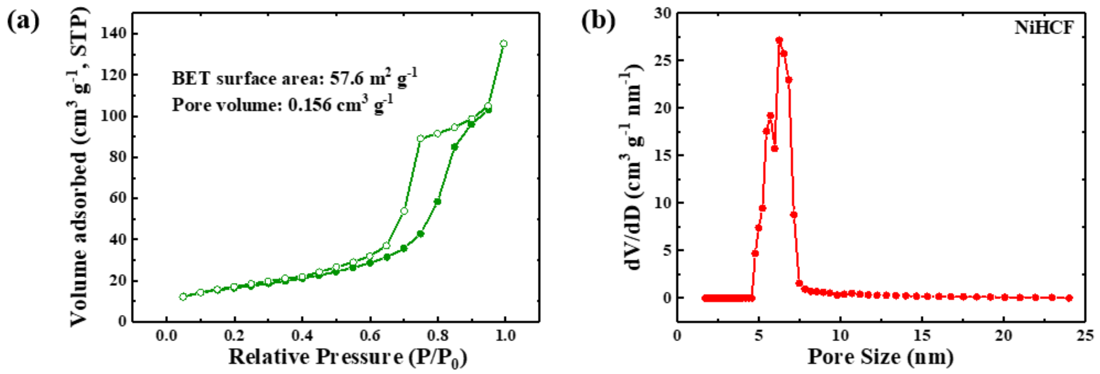

Figure S2. Nitrogen adsorption (solid circle)-desorption (empty circle) isotherms of NiHCF sample (a) and the corresponding pore size distribution (b).

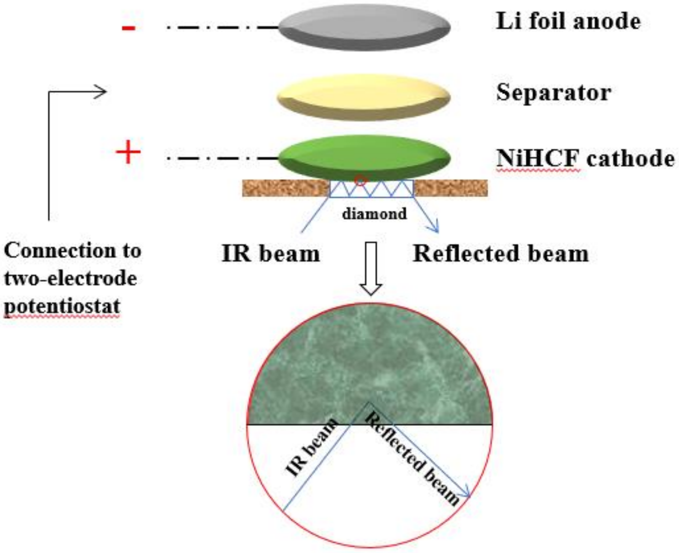

Figure S3. Cross-sectional view of the spectra-electrochemical cell combining a sandwich two-electrode design and ATR apparatus.

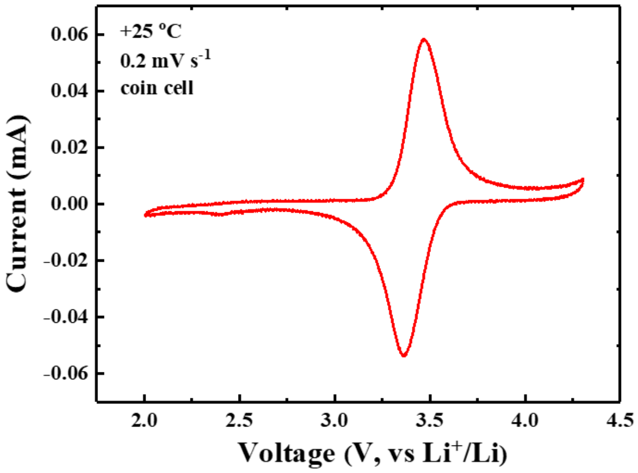

Figure S4. CV curve with the scan rate of 0.2 mV s^{-1} in the coin cell.

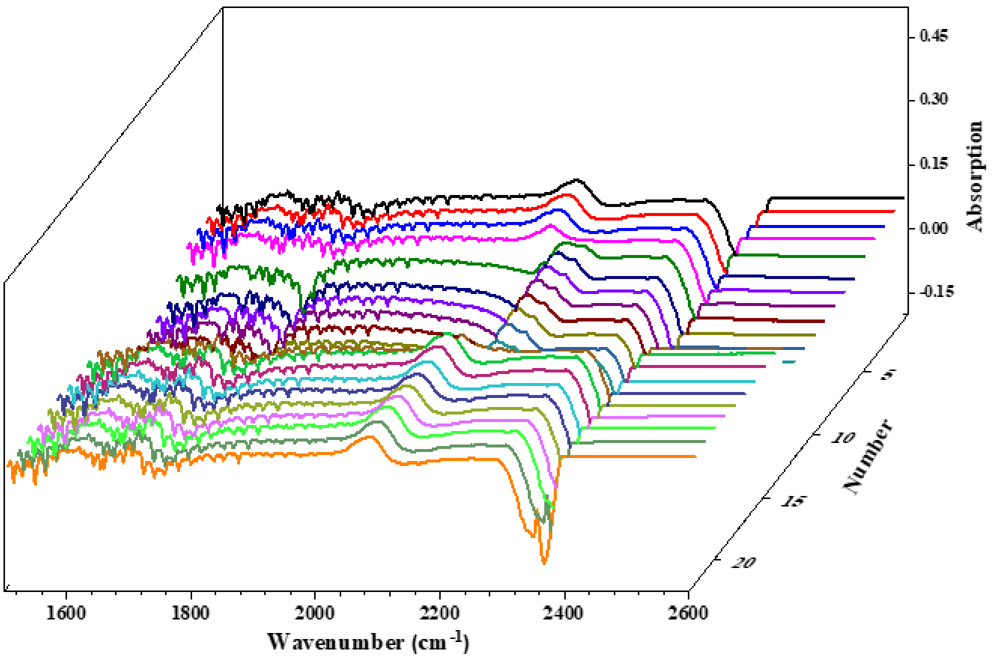

Figure S5. Difference FT-IR spectra of -CN-.

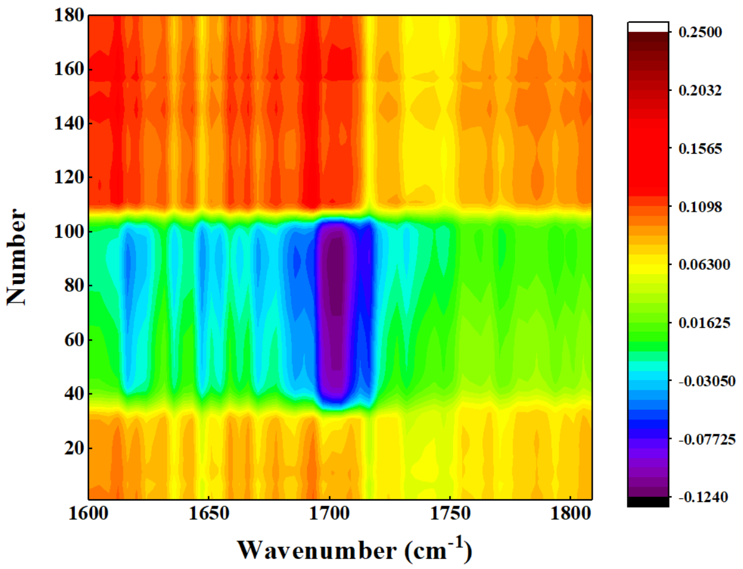

Figure S6. C=O in EA molecule during *operando* FT-IR investigation.

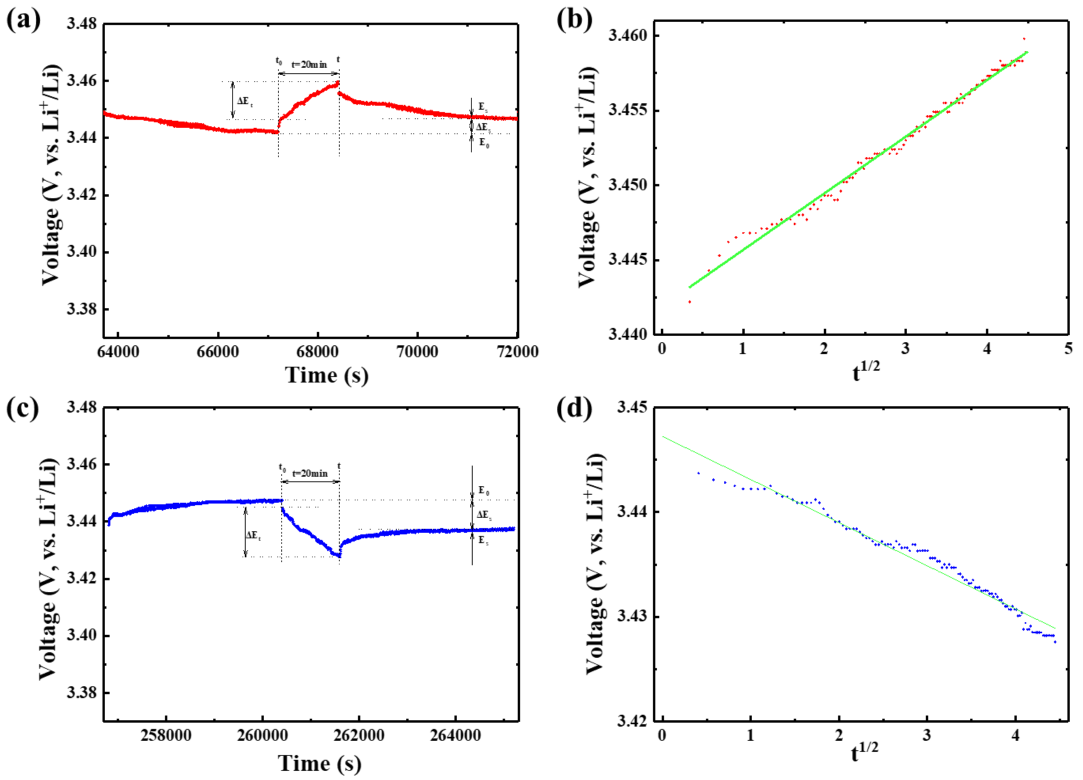

Figure S7. The determination of diffusion coefficients at +25 °C. (a) a single step of GITT to illustrate the potential response with time and (b) the representation of the transient voltage as a function of the square root of the time during charge process; (c) a single step of GITT to illustrate the potential response with time and (d) the representation of the transient voltage as a function of the square root of the time during discharge process.

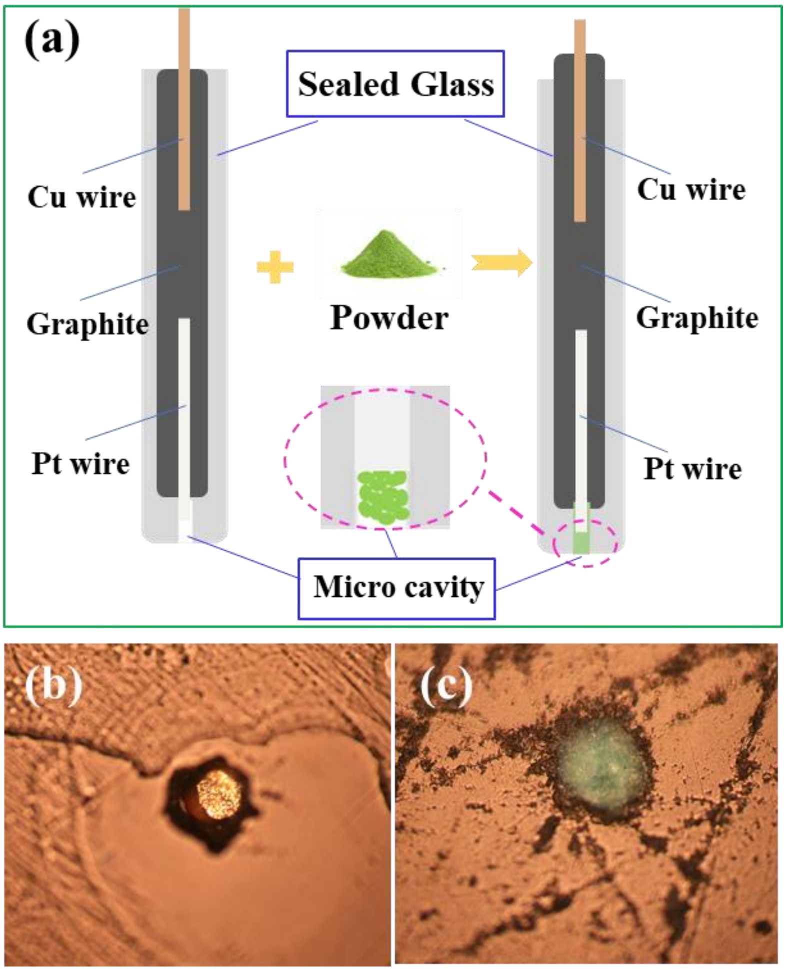

Figure S8. Schematic illustration of the cavity micro-electrode (a), optical images before (b) and after (c) loading materials.

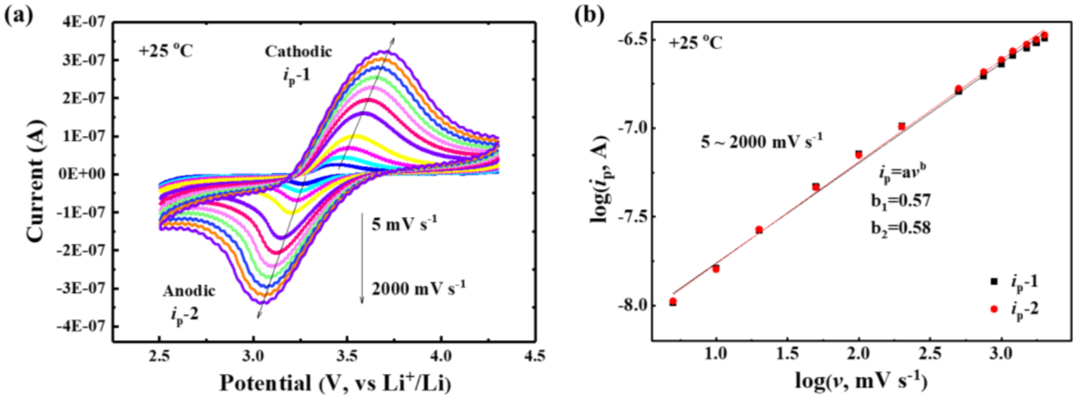

Figure S9. Kinetic analysis of the electrochemical behavior with larger sweep rates at +25°C. (a) CV curves with the scan rates from 5 mV s^{-1} to 2000 mV s^{-1}, (b) corresponding calculation of b-value.

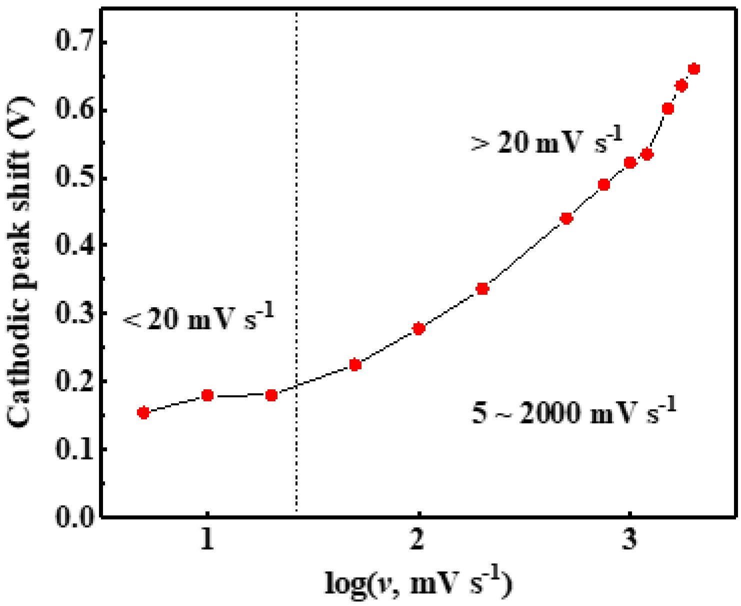

Figure S10. The variation of the cathodic peak voltage with the sweep rate. Small peak separation can be detected at sweep rate < 20 mV s^{-1}.

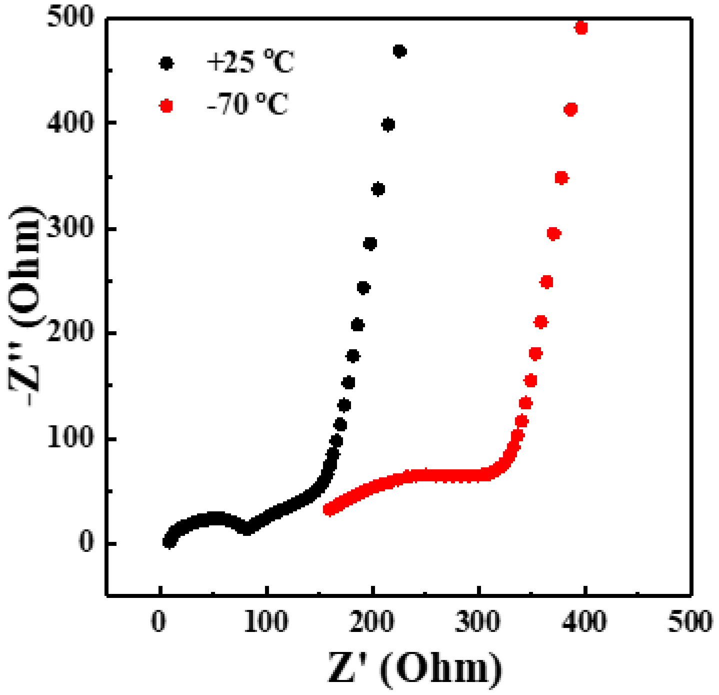

Figure S11. Electrochemical Impedance spectra (EIS) at the temperature of +25°C and -70°C.

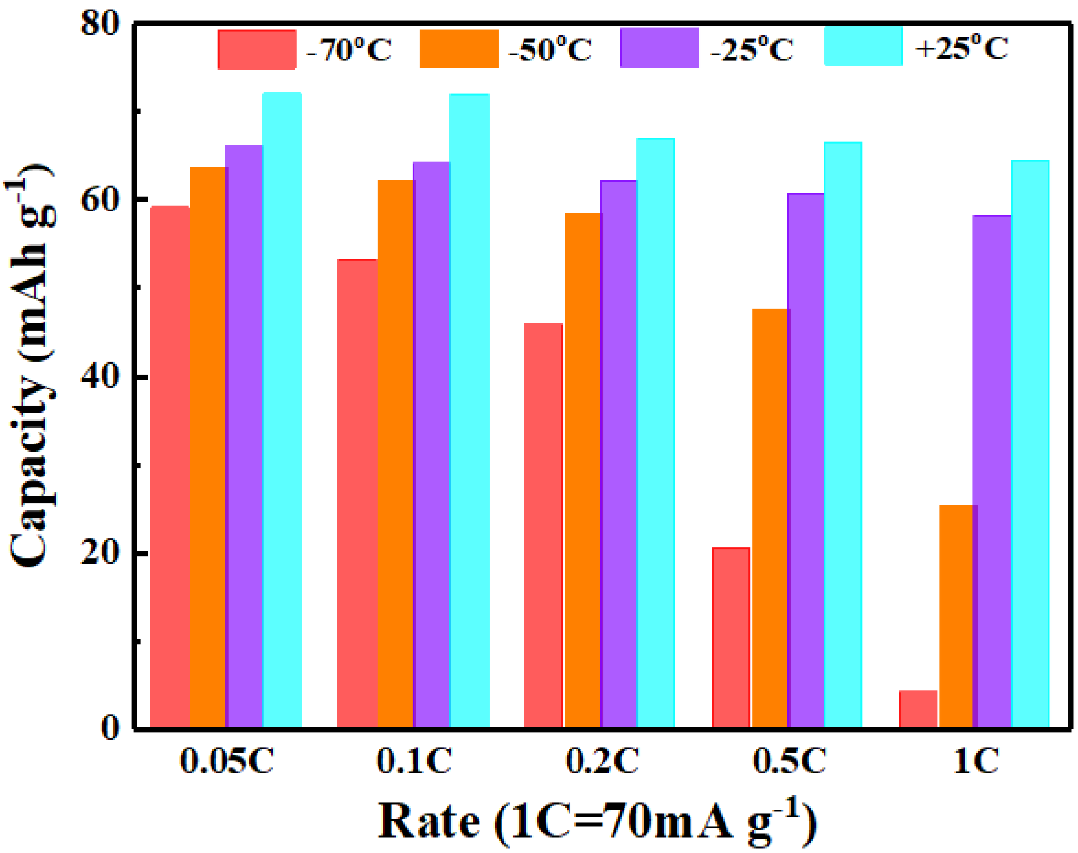

Figure S12. Rate performance at different temperatures.

Table S2 The low temperature performance compared with recent reports.

Cathode‖Anode	Temperature (°C)	C-rate	Retention (%)	Ref.
**NiHCF‖Li**	**-70**	**0.1C**	**74**	**This work**
$LiNi_{0.8}Co_{0.15}Al_{0.05}O_2$‖Li	-67	C/15	80	Nat. Energy, 2019, 4, 882-890.
LTO‖Li	-40	0.1C	80	
	-80	0.1C	60	Energy Stor. Mater., 2019, 23, 383-389.
LCO‖LTO	-50	0.1C	65	
LCO‖graphite	-60	0.1C	78.1	Angew. Chem. Int. Ed., 2019, 58, 18892-18897
Graphite‖graphite	-60	0.1C	87.3	
$LiNi_{0.5}Mn_{1.5}O_4$‖MCMB	-60	0.1C	47	J. Power Sources, 2019, 416, 29-36.
$LiCoO_2$‖Li	-10	0.1C	98.3	Science, 2017, 356, 1351.
	-60	0.1C	60.6	
$LiMn_{0.4}Fe_{0.6}PO_4$‖Li	-20	0.2C	68.7	Electrochim. Acta. 2016, 196,377-385.
$LiFePO_4$‖Li	-25	0.2C	71.4	Adv. Energy Mater., 2013, 3, 1155-1160.
$LiFePO_4$‖Li	-30	0.1C	51.5	Int. J. Electrochem. Sci., 2013, 8, 8502-8512.
$LiFePO_4$‖Li	-40	0.3C	38.9	Electrochim. Acta., 2012, 60, 269-273.
$LiNiCoO_2$‖MCMB	-40	C/16	76.53	J. Electrochem. Soc., 2012, 159(6), A739-751.
	-50	C/16	42.69	
$LiNiCoO_2$‖MCMB	-50	C/16	61%	J. Electrochem. Soc., 2010, 157(12), A1361-1374
	-60	C/16	29%	

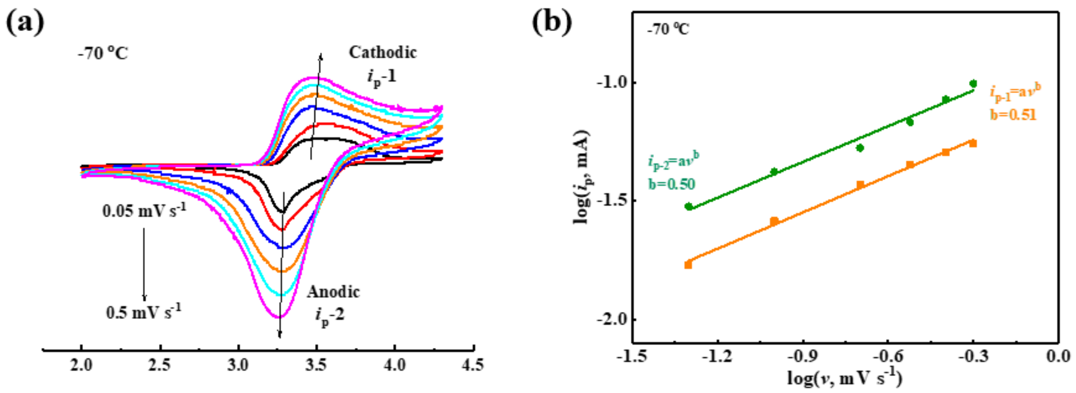

Figure S13. Kinetic analysis of the electrochemical behavior at -70°C. (a) CV curves with the scan rates from 0.05 mV s^{-1} to 0.5 mV s^{-1}, (b) corresponding calculation of b-value.

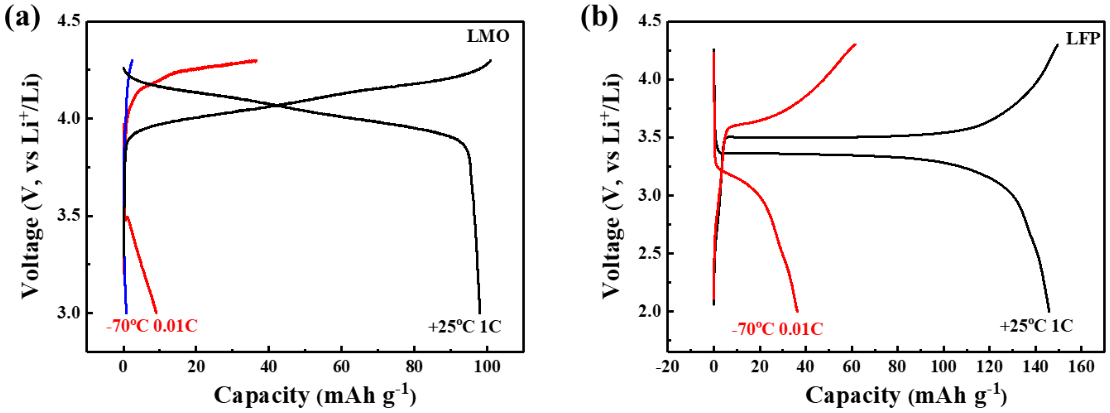

Figure S14. Low temperature performance of commercialized intercalation compounds LMO (a) and LFP (b).

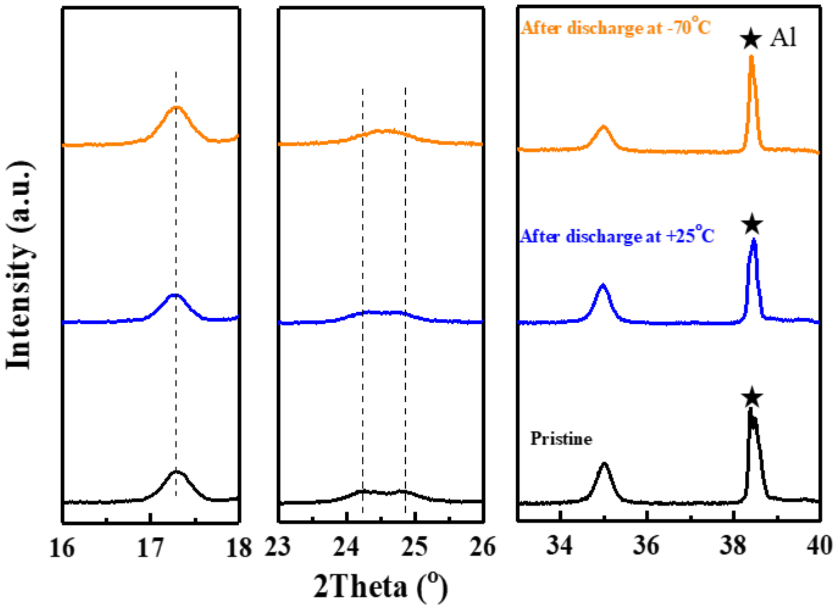

Figure S15. Ex-situ XRD investigations after discharge at different temperatures. All the batteries are discharged to a certain capacity of 30 mAh g^{-1} to control same state of charge.

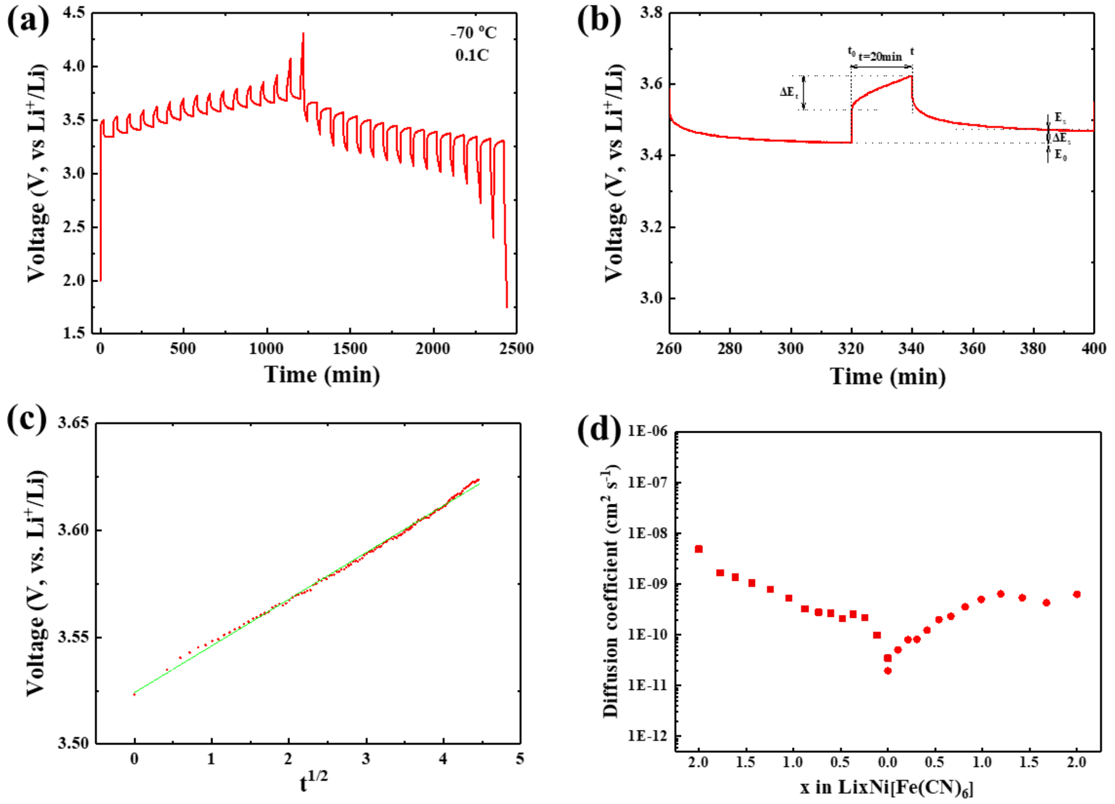

Figure S16. The determination of diffusion coefficients at -70°C. (a) GITT curves for charge/discharge, (b) a single step of GITT to illustrate the potential response with time, (c) the representation of the transient voltage as a function of the square root of the time, (d) corresponding diffusion coefficients upon delithiation/lithiation.

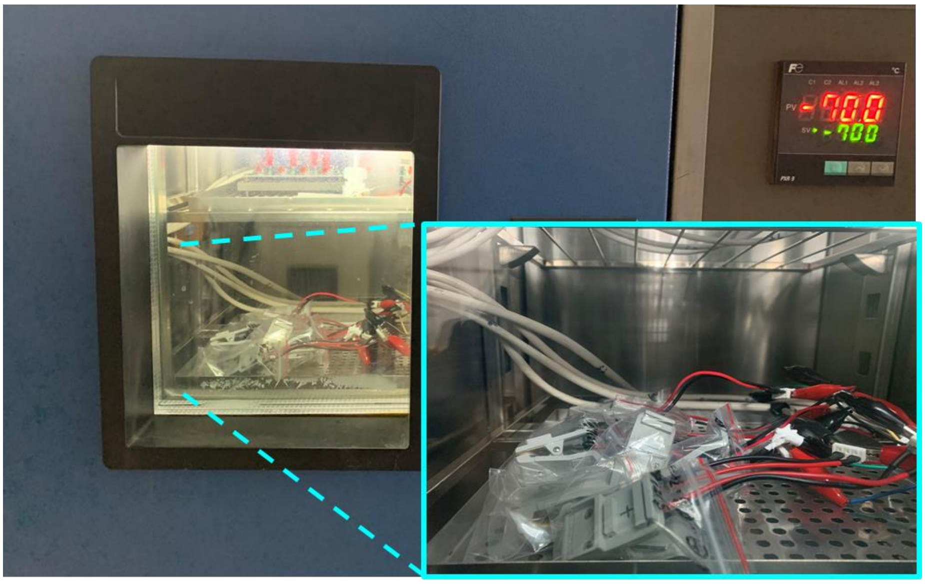

Figure S17. Optical image of the low temperature battery test in the high-low temperature test chamber connected with a charge/discharge instrument. The operating temperature range is from -75°C to 100°C.
